# Clinical parameters predicting failure of empirical antibacterial therapy in early onset neonatal sepsis, identified by classification and regression tree analysis

**DOI:** 10.1186/1471-2431-9-72

**Published:** 2009-11-24

**Authors:** Tuuli Metsvaht, Heti Pisarev, Mari-Liis Ilmoja, Ülle Parm, Lea Maipuu, Mirjam Merila, Piia Müürsepp, Irja Lutsar

**Affiliations:** 1 Paediatric Intensive Care Unit, Clinic of Anaesthesiology and Intensive Care, Tartu University Clinics, Lunini 6, 51014 Tartu, Estonia; 2 Institute of Public Health, Tartu University, Ravila 19, 50411 Tartu, Estonia; 3 Paediatric Intensive Care Unit, Tallinn Children's Hospital, Tervise 28, 13419 Tallinn, Estonia; 4 Institute of Microbiology, Tartu University, Ravila 19, 50411 Tartu, Estonia; 5 Department of Paediatrics, Tartu University Clinics, Lunini 6, 51014 Tartu, Estonia

## Abstract

**Background:**

About 10-20% of neonates with suspected or proven early onset sepsis (EOS) fail on the empiric antibiotic regimen of ampicillin or penicillin and gentamicin. We aimed to identify clinical and laboratory markers associated with empiric antibiotic treatment failure in neonates with suspected EOS.

**Methods:**

Maternal and early neonatal characteristics predicting failure of empiric antibiotic treatment were identified by univariate logistic regression analysis from a prospective database of 283 neonates admitted to neonatal intensive care unit within 72 hours of life and requiring antibiotic therapy with penicillin or ampicillin and gentamicin. Variables, identified as significant by univariate analysis, were entered into stepwise multiple logistic regression (MLR) analysis and classification and regression tree (CRT) analysis to develop a decision algorithm for clinical application. In order to ensure the earliest possible timing separate analysis for 24 and 72 hours of age was performed.

**Results:**

At 24 hours of age neonates with hypoglycaemia ≤ 2.55 mmol/L together with CRP values > 1.35 mg/L or those with BW ≤ 678 g had more than 30% likelihood of treatment failure. In normoglycaemic neonates with higher BW the best predictors of treatment failure at 24 hours were GA ≤ 27 weeks and among those, with higher GA, WBC ≤ 8.25 × 10^9 ^L^-1 ^together with platelet count ≤ 143 × 10^9 ^L^-1^. The algorithm allowed capture of 75% of treatment failure cases with a specificity of 89%. By 72 hours of age minimum platelet count ≤ 94.5 × 10^9 ^L^-1 ^with need for vasoactive treatment or leukopaenia ≤ 3.5 × 10^9 ^L^-1 ^or leukocytosis > 39.8 × 10^9 ^L^-1 ^or blood glucose ≤ 1.65 mmol/L allowed capture of 81% of treatment failure cases with the specificity of 88%. The performance of MLR and CRT models was similar, except for higher specificity of the CRT at 72 h, compared to MLR analysis.

**Conclusion:**

There is an identifiable group of neonates with high risk of EOS, likely to fail on conventional antibiotic therapy.

## Background

The role of early adequate antibacterial (AB) therapy in reducing mortality of serious infections in adult intensive care setting has been well recognised [1] and has led to implementation of broad spectrum agents as a primary choice in high risk situations. In neonatal care the present use of empirical antibiotics has not allowed similar approach, as only about 2-4% of all neonates receiving it, finally develop proven serious infections [2,3]. In addition, widespread use of broad spectrum agents carries the potential hazard of increasing antimicrobial resistance and probably even mortality [4-6]. However, in the era of the escalating role of Gram-negative bacteria in neonatal early onset sepsis (EOS) and spreading antibiotic resistance among community acquired strains [2,7] immediate implementation of broad spectrum coverage in a selected population of neonates with high risk of treatment failure might be justified. Neonatal sepsis is a complex disease with variable clinical presentation and severity. The routine interpretation of clinical and laboratory parameters is generally of little help in distinguishing between patients infected with antibiotic resistant and susceptible microorganisms. Furthermore blood cultures are often negative and their results will be available after 24 h the earliest [8,9]. To our best knowledge no study has identified clinical and laboratory markers associated with AB treatment failure in proven or suspected EOS.

Classification and regression tree (CRT) analysis has gained increasing popularity as a method for clinical decision rule construction due to its proven ability of outcome prediction [10] and relatively easy application of the results in everyday clinical practice [11]. The analysis combines the advantages of statistical approach (regression analysis) and data mining techniques (decision tree) for generation of easily interpretable rules for clinical decision making [12]. The aim of the present study was to identify perinatal and early neonatal factors that could predict failure of empiric AB regimen of ampicillin or penicillin with gentamicin, in neonates with high suspicion of EOS.

## Methods

### Study design and empirical antibiotic treatment

A post-hoc analysis of a prospective database of an open label cluster-randomised study conducted in two third level neonatal intensive care units (NICU) in Estonia from August 2, 2006 to November 30, 2007 was performed. The study aimed to compare the clinical efficacy of ampicillin and gentamicin to that of penicillin G and gentamicin in the empirical treatment of EOS. The details of the study design have been described elsewhere [13]. As no difference between the clinical efficacy of the empirical antibiotic regimens was detected, the data of patients in both study arms were pooled.

### Patients

The study included all neonates admitted within the first 72 h of life, needing antibiotic treatment with ampicillin or penicillin combined with gentamicin for suspected EOS or due to risk factors of infection according to the CDC criteria (e.g. maternal chorioamnionitis and/or maternal risk factors of infection and/or preterm labour in <35 weeks of gestation) [14]. The exclusion criteria were prior administration of a different antibiotic regimen for more than 24 h, transfer to another unit within the first 24 h or presence of suspected or proven meningitis, necrotizing enterocolitis (NEC), peritonitis or severe sepsis and septic shock with isolation of microorganisms resistant to the initial empiric regimen in maternal urinary tract or birth canal or other situations where the treating physician considered a different antibiotic regimen necessary.

### Data collection

The following maternal and perinatal characteristics were registered: maternal age and parity; history of spontaneous and artificial abortions; history of a neonate with group B streptococcal (GBS) disease; maternal chronic diseases, with special attention to diabetes, autoimmune diseases and malignancies; therapies used during pregnancy; drug and alcohol abuse; smoking; number of foetuses; invasive procedures during pregnancy (amniocentesis, foetal transfusions, cervical cerclage); date of maternal bacterial infections during pregnancy, including pathogens isolated from urinary tract and birth canal; type and time of AB treatment during pregnancy and delivery; premature rupture of membranes (PROM) for more than 18 h; prenatal glucocorticoid prophylaxis, divided as partial (delivery occurred in less than 24 h after the administration of the first dose) or full course (delivery occurred more than 24 h after the administration of the first dose); mode of delivery, including reasons for caesarean section (CS). The latter were further grouped as foetal if signs of acute foetal distress or medically indicated early CS for signs of foetal compromise were present or maternal in all other reasons [15,16].

Early neonatal parameters included demographic characteristics and need of intensive care interventions within the first 72 h of life as follows: birth weight (BW) and gestational age (GA); first and fifth minute Apgar score; need for respiratory support in the delivery room, age at intubation and surfactant administration, need and duration of sustained respiratory support; age on admission to NICU, time and type of initial and subsequent AB regimens, need for vasoactive therapy within the first three days of life with number of agents used; and intolerance of enteral feeding, defined as less than 10% of total calories supplied via the enteral route. If adequate enteral feeding was not tolerated, neonates received parenteral nutrition from the first day of life. Mean arterial blood pressure below the value of GA in weeks and/or signs of inadequate tissue perfusion reflected by metabolic acidosis, elevated serum lactate level served as an indication for vasoactive therapy. The following laboratory values were registered at least once a day: total blood count (TBC) including white blood cell count (WBC), differential and ratio of immature to total neutrophil count (I/T ratio), C-reactive protein (CRP); serum glucose and total bilirubin. The values of serum albumin, creatinine, urea and liver function tests (LFT) were registered for Days 2-3 only, as the Day 1 values were considered to reflect the condition of the mother rather than that of the neonate.

The diagnosis of EOS was defined as sepsis occurring within the first 72 h of life [2] and was based on at least two clinical (hyper- or hypothermia, apnea or bradycardia spells, increased oxygen requirement, feeding intolerance, abdominal distension, lethargy and hypotonia, hypotension, skin and subcutaneous lesions such as petechial rash, abscesses, sclerema) and two laboratory criteria (WBC <5000 or >20,000 × 10^9 ^cells/L; I/T ratio > 0.2; platelet count <100,000 × 10^9^/L; CRP >10 mg/L) [17]. Proven sepsis was diagnosed, when in addition to the clinical and laboratory signs of sepsis a true pathogen (except coagulase-negative staphylococci (CoNS) that had to be isolated from at least two different specimens or with only one positive culture adequate antibiotic treatment had to be given for more than 72 hours) was isolated from a normally sterile body fluid [18], all other cases were termed clinical sepsis. Autopsy with histological investigations to look for evidence of inflammation was conducted in all deaths.

### Failure of empiric antibiotic therapy

The study protocol pre-specified the following criteria for the change of initial (in first 72 hours) antibiotics: (1) suspicion of meningitis or abdominal infection/NEC; (2) isolation of bacteria resistant to the empiric antibiotic regimen from maternal urinary tract/birth canal of a neonate with signs and symptoms of sepsis; (3) isolation of bacteria resistant to the empiric antibiotic regimen from a neonate with signs and symptoms of sepsis; (4) no improvement or deterioration of clinical findings; (5) suspicion of nosocomial infection and (6) other situations where the treating physician considered change of antibiotic regimen necessary [13]. In the comparison of ampicillin plus gentamicin vs penicillin plus gentamicin in the empiric treatment of EOS, all cases where AB treatment was changed within 72 h and all early neonatal deaths were considered treatment failures. For the purposes of this analysis a further review was conducted by two of us (TM and IL) taking into account final clinical and autopsy data. Only cases with clear evidence of infection were included and they formed a group of empiric antibiotic therapy failure, divided as follows: (1) isolation of EOS etiologic pathogen(s), resistant to initial therapy, from normally sterile body fluids; (2) diagnosis of clinical EOS and death with autopsy confirming congenital infection (polymorphonuclear infiltration of multiple tissues with or without bacteria on Gram-stain and microbiological cultures positive for a known neonatal pathogen) or clinical EOS and major sepsis-related complication (i.e. intraventricular haemorrhage grade III-IV) within the first week of life; (3) change of empirical AB therapy within 72 h due to deterioration of EOS with the new regimen continued for more than 72 h.

### Statistical analysis

Statistical analysis was performed using statistical software R 2.7.2 http://www.r-project.org/. To identify predictors of treatment failure from maternal and neonatal characteristics and laboratory tests univariate logistic regression analysis was applied. If both high and low levels of a laboratory test would be considered abnormal (i.e. WBC, blood glucose), generally accepted normal limits were applied to test the possible predictive potential of a given parameter. For laboratory values indicative of sepsis the following cut-off levels were applied: WBC <5000 or >20,000 × 10^9 ^cells/L; I/T ratio > 0.2; platelet count <100,000 × 10^9^/L; CRP >10 mg/L [17]. For blood glucose values between 3.0 and 5.5 mmol/L were used as normal limits [19].

In order to find the best combination of predictors for antibiotic treatment failure, all parameters significant at a p value of ≤ 0.05 in univariate logistic regression analysis were entered into multiple logistic regression (MLR) analysis with backward stepwise removal of parameters in order of insignificance. CRT analysis was performed with the function *rpart *of package R [20]. Recursive partitioning with binary cut of entered variables was used for the decision tree development and the best separator of treatment failure and success was chosen for tree root. The same process was repeated until the best discrimination between treatment success and failure was achieved or until the minimum allowed number of seven patients per node was met. For tree pruning the k-fold cross-validation was applied. Surrogate splitting was used to handle missing values. To ensure the earliest possible timing of decisions separate MLR and CRT analysis of data available by 24 and 72 h of age was performed. For laboratory tests the highest and lowest values within 72 h were incorporated as continuous variables to allow generation of splitting values with maximum information gain. Specificity, sensitivity, positive and negative predictive values were calculated for each prediction.

The study was approved by the Ethics Committee of the University of Tartu.

## Results

The study included 283 patients; 142 were treated with ampicillin and 141 with penicillin, both in combination with gentamicin. Another 29 neonates were considered to require antibiotic therapy, different from the study regimen, on admission - 18 received preoperative antibacterial prophylaxis with cefazolin; four had suspected NEC and/or peritonitis and received metronidazole, ampicillin-sulbactam and/or piperacillin-tazobactam; three received cefotaxime for suspected meningitis and one for severe renal failure; one neonate received fluconazole treatment for candidiasis. Of the study population 79% were born in the same regional centres, participating in the study, with transfer times ranging from 10-20 minutes; the remaining 21% were out-born, transported by a specialised neonatal transport team from county hospitals. Basic demographic and clinical characteristics of the study population are shown in Table 1. Neonates who failed on initial empiric AB treatment had significantly lower GA and BW, were more often born to mothers with chorioamnionitis, had lower first and fifth minute Apgar scores, required more often surfactant therapy, were more likely to have proven EOS and to die within 7 days than those with AB treatment success. A total of 14 cases of EOS were bacteriologically proven: *Streptococcus agalactiae *- 4; *Staphylococcus epidermidis *- 3, *Staphylococcus haemolyticus *- 1, *Escherichia coli *- 2, *Enterobacter cloacae *- 2, *Haemophilus influenzae *- 1, *Candida albicans *- 1. In all 3 cases of *S. epidermidis *sepsis the diagnosis was based on at least 2 positive blood cultures not more than 72 h apart. The only case of *S. haemolyticus *EOS was based on a single positive blood culture in a neonate with clinical and laboratory signs of sepsis (CRP 151 mg/l, I/T ratio 0.65); unfortunately no further blood cultures were taken from the baby within the next 72 h. Twenty four neonates died within the first week of life (14 of infectious and 10 of non-infectious causes); autopsy was performed in all cases. Among the 10 deaths, not related to infection, six subjects had respiratory distress and died of respiratory failure or of complications of prematurity; autopsy results and post-mortem cultures were not suggestive of infection in any case; four neonates had major congenital malformations: severe pulmonary hypoplasia, complex cardiac malformation, oesophageal atresia with tracheo-oesophageal fistula (died of mediastinitis after surgical repair) and lethal dwarfism.

**Table 1:** Demographic and main clinical characteristics

	AB success N = 251	AB failure N = 32	OR (95% CI) or p-value
Gestational age (weeks) - mean (SD)	31.7 (5.1)	28.8 (4.4)	**0.0034**

>36 weeks - n (%)	55 (21.9)	2 (6.3)	0.24 (0.06-1.03)

<28 weeks - n (%)	61 (24.3)	14 (43.8)	**2.42 (1.14-5.16)**

<26 weeks - n (%)	35 (13.9)	10 (31.3)	**2.81 (1.23-6.42)**

Birthweight (g) - mean (SD)	1913 (1086)	1350 (978)	**0.0065**

<1501 g - n (%)	122 (48.6)	23 (71.9)	**2.70 (1.20-6.07)**

<1001 g - n (%)	59 (23.5)	16 (50.0)	**3.25 (1.53-6.90)**

<751 g - n (%)	23 (9.2)	11 (34.4)	**5.19 (2.23-12.10)**

M/F sex - n	143/108	20/12	1.26 (0.59-2.69)

Apgar score at 1 min - mean (SD)	5.3 (2.1)	4.3 (1.9)	**0.0114**

Apgar score at 5 min - mean (SD)	6.5 (1.5)	5.9 (1.5)	**0.0422**

Ventilated - n (%)	187 (74.5)	28 (87.5)	2.40 (0.81-7.09)

Surfactant - n (%)	141 (56.2)	28 (87.5)	**5.46 (1.86-16.03)**

Cesarean section - n (%)	142 (56.6)	15 (46.9)	0.68 (0.32-1.42)

Multiple pregnancies - n (%)	50 (19.9)	7 (21.9)	1.13 (0.46-2.75)

Chorioamnionitis - n (%)	40 (15.9)	11 (34.4)	**2.76 (1.24-6.17)**

PROM >18 h - n (%)	46 (18.3)	7 (21.9)	1.25 (0.51-3.06)

Prenatal glycocorticoids (full course) - n (%)	106 (42.2)	8 (25.0)	0.46 (0.20-1.05)

AB therapy during pregnancy	61 (24.3)	4 (12.5)	0.45 (0.15-1.32)

AB therapy during delivery	91 (36.3)	6 (18.8)	0.41 (0.16-1.02)

Proven EOS - n (%)	4 (1.6)	10 (31.3)	28.07 (8.13-96.88)

Early neonatal mortality - n (%)	10 (4.0)	14 (43.8)	18.74 (7.31-48.10)

OR - odds ratio; 95% CI - 95% confidence interval; PROM - premature rupture of membranes; EOS - early onset neonatal sepsis; AB - antibiotic

### Failure of empiric antibiotic therapy

A total of 32 neonates fulfilled the criteria of AB treatment failure, 21 of whom had culture and/or histologically proven sepsis (Table 2). Two of 10 resistant microorganisms causing EOS (*S. haemolyticus *and *Candida albicans*) were resistant to both and eight to one component of the corresponding empiric antibiotic treatment (*E. coli *and *E. cloacae *to ampicillin and 3 strains of *S. epidermidis, E. coli, E. cloacae *and *H. influenza *to penicillin; both *E. coli *and *E. cloacae *strains were susceptible to gentamicin); AB therapy was changed within 72 h in two cases. Among the 14 cases of early neonatal death due to infection, four had culture proven EOS and the remaining 10 had histological changes suggestive of infection. Another eleven neonates with the diagnosis of clinical EOS had AB treatment changed within the first 72 h due to deteriorating clinical condition (n = 4) or suspected meningitis (n = 7); with the new treatment regimen started at a median age of 46 (range 19-62) hours. Among the seven neonates, who were assigned to a new antibiotic regimen for suspected meningitis, in one the diagnosis was confirmed by cerebrospinal fluid laboratory results (bacterial aetiology was not identified) and the other six had the diagnosis of clinical EOS. None of these infants died, but in all seven cases new antibiotic regimen was continued for a full course of 10-14 days and all these cases were included into treatment failure group based on the criterion III (Table 2). Altogether in nine cases antibiotic regimen was changed because of deteriorating clinical condition; four died of clinical EOS, confirmed by autopsy (treatment failure criterion II in Table 2) and one of EOS due to *Candida albicans*, isolated from blood (treatment failure criterion I in Table 2). The remaining four neonates had the final diagnosis of clinical EOS and recovered after a 7-10 day course of the new AB regimen (treatment failure criterion III in Table 2).

**Table 2:** Distribution of patients with empiric antibiotic treatment failure

Failure of early empirical AB regimen - N (% of all study patients)	32 (11.6)
I Proven EOS with etiologic pathogen(s) resistant to initial empirical AB therapy - n (% of treatment failure)	10 (31.3)

died within 7 days	4

II clinical EOS and death (with autopsy confirming the diagnosis of congenital infection) and/or major sepsis-related complication (IVH III-IV) within the first week of life - n (% of treatment failure)	11^a ^(34.4)

died within 7 days	10

III clinical EOS and change of initial empirical AB therapy within 72 h with new regimen continued for more than 72 h - n (% of treatment failure)	11 (34.4)

All cases are represented once - in the "highest" applicable category, i.e. a neonate with culture positive sepsis with a pathogen, resistant to empiric regimen is included only in proven EOS category, independent of whether he died within 7 days or not. ^a ^includes an ELBW neonate with clinical EOS and IVH IV by the third day of life who died on Day 8, autopsy confirmed the diagnosis of EOS. AB - antibiotic; EOS - early onset sepsis; IVH - intraventricular haemorrhage

### Univariate and multiple logistic regression and classification and regression tree analysis

Univariate logistic regression analysis identified a total of 21 factors (4 maternal, 6 neonatal and 14 laboratory parameters) as predictors of treatment failure (Table 3). Complete set of data for MLR was available for 201/251 (80%) treatment success and 31/32 (97%) treatment failure cases at 24 hours and for 252 (89%; 222 cases of treatment success and 30 cases of treatment failure) at 72 hours; the factors predicting failure are presented in Table 4. The four parameters (need for vasoactive treatment, WBC <5000 or >20000 per mm^3^, I/T ratio >0.2 and platelet count) identified by MLR at 24 hours predicted treatment failure and success correctly in 19/31 (sensitivity 61%) and 180/201 cases (specificity 90%), respectively (Table 5). Among predictors of treatment failure at 24 hours only need for vasoactive treatment and platelet count, remained significant also at 72 hours (Table 4). The 72 hour model predicted treatment failure and success correctly in 24/30 (sensitivity 80%) and 173/222 cases (specificity 78%), respectively.

**Table 3:** Significant predictors of empiric antibiotic failure: results of univariate logistic regression analysis

	OR (95% CI)	p-value
Maternal and perinatal characteristics		

Maternal chorioamnionitis (yes vs. no)	2.76 (1.24-6.17)	0.0132

Maternal smoking during pregnancy (yes vs. no)	3.13 (1.05-9.38)	0.041

Isolation of a pathogen from maternal urinary tract; birth canal or placenta during delivery	6.39 (1.36-29.97)	0.0187

Neonatal characteristics		

1^st ^minute Apgar score (per 1 point increase)	0.79 (0.66-0.95)	0.0124

Birthweight (per 100 g increase)	0.94 (0.89-0.98)	0.008

Gestational age (per week increase)	0.88 (0.81-0.96)	0.004

Age on admission to ICU (per hour increase)	0.84 (0.70-0.99)	0.0411

Number of vasoactive drugs (per 1 additional drug)	2.50 (1.76-3.54)	0.000

Intolerance of enteral feeds within 3 days	2.64 (1.17-5.92)	0.0188

Laboratory characteristics		

WBC <5000 or >20000 per mm^3 ^on Day 1	3.42 (1.61-9.47)	0.0014

WBC <5000 or >20000 per mm^3 ^on Day 2-3	2.84 (1.33-6.10)	0.0072

I/T ratio >0.2 on Day 1	3.90 (1.61-9.47)	0.0026

I/T ratio >0.2 on Day 2-3	4.25 (1.55-11.62)	0.0048

Platelet count on Day 1 (per 10,000 mm^-3 ^increase)	0.89 (0.83-0.95)	0.0003

Platelet count on Day 2-3 (per 10,000 mm^-3 ^increase)	0.86 (0.81-0.92)	0.0000

Haemoglobin on Day 2-3 (per 10 g/L increase)	0.72 (0.60-0.86)	0.0002

C-reactive protein > 10 mg/l on Day 1	3.34 (1.39-8.01)	0.007

C-reactive protein > 10 mg/l on Day 2-3	2.53 (1.17-5.45)	0.0179

Blood glucose < 3.0 mmol/l or >5.5 mmol/l on Day 1	4.28 (1.45-12.64)	0.0084

Blood glucose < 3.0 mmol/l or >5.5 mmol/l on Day 2-3	3.72 (1.55-8.97)	0.0034

Urea on Day 2-3 (per 1 mmol/L increase)	1.08 (1.00-1.16)	0.0411

Serum albumin on Day 2-3 (per 1 g/L increase)	0.82 (0.76-0.90)	0.0000

Serum AST on Day 2-3 (per 1 IU/L increase)	1.00 (1.00-1.00)	0.0231

OR - odds ratio; 95% CI - 95% confidence interval; ICU - intensive care unit; WBC - white blood cell count; I/T ratio - immature to total neutrophil ratio; AST - aspartate aminotransferase

**Table 4:** Multiple logistic regression analysis of clinical and laboratory variables predicting failure of empiric antibiotic regimen at 24 and 72 hours of age

	OR (95% CI)	p-value
24 hour model (n = 232)		
Need for vasoactive treatment	2.83 (1.21-6.66)	0.0167
WBC <5000 or >20000 per mm^3 ^on Day 1	2.51 (1.09-5.81)	0.0308
I/T ratio >0.2 on Day 1	2.79 (1.10-7.11)	0.0312
Platelet count on Day 1 (per 10,000 mm^-3 ^increase)	0.92 (0.86-0.98)	0.0124

72 hour model (n = 252)		

Need for vasoactive treatment	4.43 (1.55-12.68)	0.0055
Platelet count on Day 2-3 (per 10,000 mm^-3 ^increase)	0.92 (0.86-0.99)	0.0331
C-reactive protein (per 1 mg/l increase) on Day 1	1.02 (1.00-1.03)	0.0359
Serum albumin on Day 2-3 (per 1 g/L increase)	0.87 (0.80-0.95)	0.0029

OR - odds ratio; 95% CI - 95% confidence interval; ICU - intensive care unit; WBC - white blood cell count; I/T ratio - immature to total neutrophil ratio

**Table 5:** Diagnostic value of models for prediction of empiric antibiotic treatment failure in neonates.

	MLR analysis		Classification and regression tree analysis	
	**24 h model**	**72 h model**	**24 h model**	**72 h model**

Sensitivity (%)	61	80	75	81

Specificity (%)	90	78	89	88*

Positive predictive value (%)	48	33	46	46

Negative predictive value (%)	94	97	97	97

* difference from MLR model of the same time point significant at p < 0.05

MLR - multiple logistic regression

**Figure 1:**
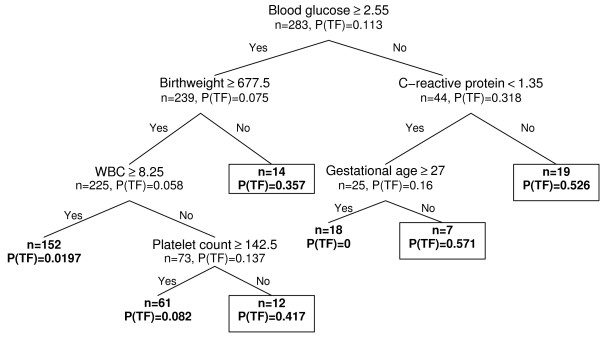
**Decision tree based on data available by 24 h from birth with patient distribution**. P(TF) - probability of treatment failure. For laboratory values the decision algorithm includes the highest and/or lowest values registered within the first 24 h of life. Patient groups with P(TF) ≥ 0.3, identified as treatment failures, are surrounded by black box. WBC - white blood cell count.

**Figure 2:**
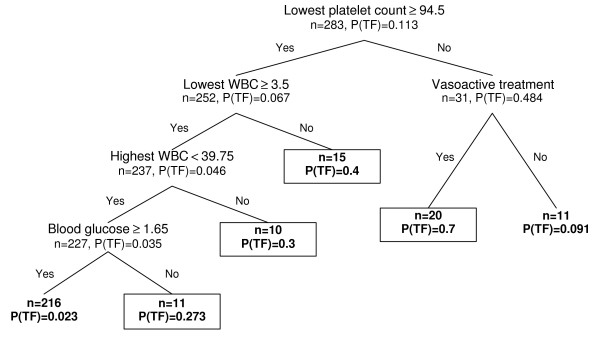
**Decision tree based on data available by 72 h from birth with patient distribution**. P(TF) - probability of treatment failure. For laboratory values the decision algorithm includes the highest and/or lowest values registered within the first 72 h of life. Patient groups with P(TF) > 0.27, identified as treatment failures, are surrounded by black box. WBC - white blood cell count.

The CRT model, as created through recursive partitioning of parameters available by 24 h of age and the distribution of patients through the model, is shown in Figure 1. The principal discriminator was hypoglycaemia ≤ 2.55 mmol/L which was followed by CRP values > 1.35 mg/L in infants with hypoglycaemia and by BW ≤ 678 g in those without. Further partitioning was based on GA ≤ 27 weeks or WBC ≤ 8.25 × 10^9 ^L^-1 ^together with platelet count ≤ 143 × 10^9 ^L^-1^. Applying antibiotic failure probability of 0.3 as a cut-off limit the algorithm allowed capture of 75% of treatment failure cases (24/32; 95% CI 56-87%) with a specificity of 89% (223/251; 95% CI 84-92%). The model using data available by 72 h of age together with the distribution of patients is shown in Figure 2. Compared to data available by 24 h, platelet count ≤ 94.5 × 10^9 ^L^-1 ^was identified as the principal discriminator, followed by the need for vasoactive treatment or WBC < 3.5 × 10^9 ^L^-1^. Further discriminators identified by the software and the corresponding cut-off values were WBC > 39.8 × 10^9 ^L^-1 ^and blood glucose ≤ 1.65 mmol/l, respectively. Incorporating 72 h data to CRT model lowered the cut-off probability of treatment failure to 0.27 and the performance of the algorithm improved to a sensitivity of 81% (26/32; 95% CI 63-92%) and a specificity of 88% (221/251; 95% CI 83-91%). A total of 36 cases were misclassified (6 AB failure and 30 success cases), resulting in an overall accuracy of 87%.

The comparative performance of MLR and CRT models is shown in Table 5. Both statistical approaches achieved high negative predictive values at 24 and 72 h of life; however the positive predictive value (PPV) remained below 50% in all predictions. At 72 h the CRT model achieved higher specificity than MLR analysis.

## Discussion

To our knowledge this is the first study attempting to identify clinical characteristics, predictive of empirical antibiotic treatment failure in neonates with high suspicion of EOS. We suggest two clinical decision algorithms that allow prediction of AB treatment failure with a sensitivity of 75-81% and a specificity of 88-89% by 24 and 72 h of age, respectively. Using CRT analysis we have identified cut-off limits for early clinical parameters of uncontrolled sepsis, like WBC, blood glucose and platelet count, allowing, when combined with GA and BW data, capture of 75% of treatment failure cases with a specificity of 89% within 24 h.

CRT analysis was chosen, as more traditional methods, like MLR are often cumbersome or of limited utility for bedside application [21,22]. The decision algorithms resulting from CRT analysis with the graphical presentation, on the contrary, are easy to interpret and apply in clinical practice. This analysis is inherently non-parametric, so that no assumptions are made regarding the underlying distribution of variables and thus categorical as well as continuous parameters can be used. In CRT analysis the data are allowed to determine which clinical variables (as well as the corresponding cut-offs for continuous variables) are applied [23]. The order and use of variables are derived by the software without any bias other than the choice of potential predictors. In order to avoid any investigator induced bias all maternal, early neonatal and laboratory parameters identified by univariate regression analysis as significant predictors of treatment failure, were included.

The performance of the MLR models as well as the CRT algorithms is similar to those, described for identification of blood stream infections in febrile patients [23,24]. However, compared with MLR, CRT analysis achieved higher sensitivity in identification of patients who fail on initial AB regimen at 24 hours and higher specificity by 72 hours of age, even with the relatively low cut-off probability of treatment failure (0.27-0.30). The latter ensured higher sensitivity, as inadequate AB therapy carries high risk for the patient. Nevertheless, a high negative predictive value was preserved for both analyses, which is of equal importance in order to avoid unnecessary AB exposure. In consistency with the time course of disease the model used slightly different choice of parameters and cut-off values depending on the evaluated time point; not surprisingly a more profound deviation from normal values was detected by 72 h, compared to 24 h of age.

Although all parameters identified for the decision algorithms have been used as diagnostic markers of EOS earlier [25] this analysis allowed to identify cut-off values for AB treatment failure, as determined by the data, at well defined time points. The fact that all included parameters are readily available in most NICUs enhances the clinical applicability of the decision tree considerably. Hypoglycaemia has been suggested as a predictor of adverse outcome in neonatal infections [26]. Still, we have identified different cut-off levels for blood glucose at 24 and 72 h of age, with lower levels applied with advancing age. The 72 h blood glucose level of less than 1.65 mmol/l identified in this study is somewhat lower compared to 2.4 mmol/l shown to be associated with adverse outcome of neonatal septic shock in a recent study by Kermorvant [26]. Similar to Bachur and co-authors who used CRT analysis in febrile infants for identification of serious bacterial infections [23] we found that not neutrophil count or I/T ratio but the total WBC, with the cut-off values of <8.25 × 10^9 ^L^-1 ^at 24 h and <3.5 × 10^9 ^L^-1 ^or >39.8 × 10^9 ^L^-1 ^at 72 h of age, has the best discriminative power in prediction of AB treatment failure in neonates with high risk of EOS. In concordance with the results of Kermorvant and co-authors who found that thrombocytopenia is associated with adverse outcome in neonatal septic shock [26] our results show that thrombocytopenia ≤ 94.5 × 10^9 ^L^-1 ^has excellent discriminative power in infants needing concomitant vasoactive support by 72 h of age, i.e. those in shock. However, both are relatively late signs of sepsis. A significantly higher platelet count of ≤ 143 × 10^9 ^L^-1^, when present concomitantly with WBC <8.25 × 10^9 ^L^-1 ^was found to be predictive of treatment failure in suspected EOS early in the course of disease, within 24 h of age. The inclusion of low BW and GA in the 24 h model could be expected as predominance of Gram-negative EOS has been described in VLBW neonates [2,27].

We chose an approach focusing on failure of empiric AB therapy, as there is a large body of data from adult intensive care supporting the crucial role of adequate initial AB therapy in sepsis outcomes [1]. In our study about 11% of neonates with high risk of EOS failed on the presently most widely used antibiotic regimen of ampicillin or penicillin in combination with gentamicin, compared to 20% reported for ampicillin plus gentamicin in a previous study by Tessin *et al *[28]. According to our results in 65% of cases change of empirical AB therapy was a clinical decision, as clinical condition may deteriorate fast but culture results are often delayed for 36-72 h [9] and many cases of neonatal sepsis may remain culture negative with present microbiological methods either due to maternal intrapartum AB exposure [29], low bacterial load or small volumes of blood obtained for culturing [30-32]. Thus, until better diagnostic tests for neonatal EOS allowing fast discrimination between pathogens, become available for routine clinical practice [33] there is a need for clinical parameters to guide the clinician in identifying neonates at high risk of treatment failure.

One of the main limitations of this study was the use of a surrogate outcome measure of AB treatment failure. This was selected because well-defined outcome measures of EOS, like culture-proven sepsis with AB susceptibility of causative pathogens and related mortality are extremely rare, making such outcome based studies hardly manageable [6]. Furthermore, with current microbiological methods, it is likely that many cases of EOS, even those leading to death will never have positive cultures [29]. Partial overlap between the study exclusion and AB treatment change criteria is not surprising, as the clinical conditions (e.g. meningitis, NEC, septic shock caused by Gram-negative bacteria resistant to ampicillin etc) prompting these decisions are likely similar. The difference however, is that the study entry criteria were evaluated early in life, before any antibiotics had been given, whereas treatment failure criteria applied only when antibiotic treatment had been initiated. To ensure the highest possible specificity we included autopsy data as still the "golden standard" of proving clinical infection when bacteriological cultures remain negative. With this approach 2/3 of treatment failure cases were based either on positive culture or autopsy findings and the remaining cases were those that required wide-spectrum antibiotic therapy for more than 72 hours. We believe that the three clinically relevant situations selected by us serve as an adequate marker of ineffective treatment.

Although there is an identifiable group of high risk neonates who may benefit from early introduction of AB treatment with agents of wide spectrum activity this approach is not problem-free as the use of broad spectrum antibiotics has been associated with the development and spread of antibiotic resistance [5]. On the other hand, in patients with sepsis early appropriate AB coverage may outweigh the risks of broad spectrum therapy. Despite that there are no studies yet, to demonstrate whether this is true also for neonates, one could speculate that due to the relative immune suppression and inability to localise the infection, neonates are even more sensitive to inappropriate AB therapy than adults.

## Conclusion

In conclusion, post-hoc analysis of a prospective database identified thrombocytopenia <94.5 × 10^9 ^L^-1 ^with concomitant need for vasoactive treatment; or WBC below 3.5 × 10^9 ^L^-1 ^or above 39.8 × 10^9 ^L^-1^; or blood glucose below 1.65 mmol/L within 72 h of life; as possible features, suggesting the failure of empiric ampicillin plus gentamicin or penicillin plus gentamicin treatment in suspected EOS. The decision algorithm, as constructed by CART analysis, needs further validation in prospective data sets. Whether implementation of broader spectrum antibiotics, based on these criteria will have any influence on the outcome of EOS remains to be answered in prospective clinical studies.

## Competing interests

The authors declare that they have no competing interests.

## Authors' contributions

TM had primary responsibility for protocol development, patient screening, enrolment, outcome assessement, preliminary data analysis, and writing the manuscript. HP participated in the development of the protocol and analytic framework for the study, was responsible for the statistical analysis and contributed to the writing of the manuscript. MLI participated in protocol development and was responsible for patient screening, enrolment and outcome assessment in one of the two participating units. ÜP, MM and PM participated in the development of the protocol and were responsible for patient screening. IL supervised the design and execution of the study, participated in the final data analyses, and contributed to the writing of the manuscript. All authors have read and approved the manuscript.

## Pre-publication history

The pre-publication history for this paper can be accessed here:

http://www.biomedcentral.com/1471-2431/9/72/prepub

## References

[B1] Garnacho-Montero J Aldabo-Pallas T Garnacho-Montero C Cayuela A Jimenez R Barroso S Ortiz-Leyba C Timing of adequate antibiotic therapy is a greater determinant of outcome than are TNF and IL-10 polymorphisms in patients with sepsis Crit Care 2006 10 4 R111 10.1186/cc4995 16859504 PMC1751000

[B2] Stoll BJ Hansen NI Higgins RD Fanaroff AA Duara S Goldberg R Laptook A Walsh M Oh W Hale E Very low birth weight preterm infants with early onset neonatal sepsis: the predominance of gram-negative infections continues in the National Institute of Child Health and Human Development Neonatal Research Network, 2002-2003 Pediatr Infect Dis J 2005 24 7 635 639 10.1097/01.inf.0000168749.82105.64 15999007

[B3] Lopez Sastre JB Fernandez Colomer B Coto Cotallo GD Ramos Aparicio A Trends in the epidemiology of neonatal sepsis of vertical transmission in the era of group B streptococcal prevention Acta Paediatr 2005 94 4 451 457 10.1080/08035250410025302 16092460

[B4] Clark RH Bloom BT Spitzer AR Gerstmann DR Empiric use of ampicillin and cefotaxime, compared with ampicillin and gentamicin, for neonates at risk for sepsis is associated with an increased risk of neonatal death Pediatrics 2006 117 1 67 74 10.1542/peds.2005-0179 16396862

[B5] de Man P Verhoeven BA Verbrugh HA Vos MC van den Anker JN An antibiotic policy to prevent emergence of resistant bacilli Lancet 2000 355 9208 973 978 10.1016/S0140-6736(00)90015-1 10768436

[B6] Mtitimila EI Cooke RW Antibiotic regimens for suspected early neonatal sepsis Cochrane Database Syst Rev 2004 4 CD004495 15495114 10.1002/14651858.CD004495.pub2 PMC8786265

[B7] Laugel V Kuhn P Beladdale J Donato L Escande B Astruc D Messer J Effects of antenatal antibiotics on the incidence and bacteriological profile of early-onset neonatal sepsis. A retrospective study over five years Biol Neonate 2003 84 1 24 30 10.1159/000071439 12890932

[B8] Berger A Witt A Haiden N Kretzer V Heinze G Pollak A Amniotic cavity cultures, blood cultures, and surface swabs in preterm infants--useful tools for the management of early-onset sepsis? J Perinat Med 2004 32 5 446 452 10.1515/JPM.2004.145 15493724

[B9] Jardine L Davies MW Faoagali J Incubation time required for neonatal blood cultures to become positive J Paediatr Child Health 2006 42 12 797 802 10.1111/j.1440-1754.2006.00980.x 17096716

[B10] Reibnegger G Weiss G Werner-Felmayer G Judmaier G Wachter H Neural networks as a tool for utilizing laboratory information: comparison with linear discriminant analysis and with classification and regression trees Proc Natl Acad Sci USA 1991 88 24 11426 11430 10.1073/pnas.88.24.11426 1763057 PMC53148

[B11] Mair J Smidt J Lechleitner P Dienstl F Puschendorf B A decision tree for the early diagnosis of acute myocardial infarction in nontraumatic chest pain patients at hospital admission Chest 1995 108 6 1502 1509 10.1378/chest.108.6.1502 7497751

[B12] Samanta B Bird GL Kuijpers M Zimmerman RA Jarvik GP Wernovsky G Clancy RR Licht DJ Gaynor JW Nataraj C Prediction of periventricular leukomalacia. Part I. Selection of hemodynamic features using logistic regression and decision tree algorithms Artif Intell Med 2009 46 3 201 15 10.1016/j.artmed.2008.12.005 19162455 PMC2745267

[B13] Metsvaht T Ilmoja M.-L Parm Ü Merila M Maipuu L Sepp E Müürsepp P Julge K Lutsar I Management of Early Onset Neonatal Sepsis: comparative study of ampicillin vs penicillin G in combination with gentamicin. ICAAC poster G2-1272 http://www.mindcull.com/images/posters/icaac2008/icaac08.G2-1272.pdf

[B14] Schrag S Gorwitz R Fultz-Butts K Schuchat A Prevention of perinatal group B streptococcal disease. Revised guidelines from CDC MMWR Recomm Rep 2002 51 RR-11 1 22 12211284

[B15] Liu S Rusen ID Joseph KS Liston R Kramer MS Wen SW Kinch R Recent trends in caesarean delivery rates and indications for caesarean delivery in Canada J Obstet Gynaecol Can 2004 26 8 735 742 15307978 10.1016/s1701-2163(16)30645-4

[B16] Joseph KS Theory of obstetrics: an epidemiologic framework for justifying medically indicated early delivery BMC Pregnancy Childbirth 2007 7 4 10.1186/1471-2393-7-4 17391525 PMC1851971

[B17] Auriti C Rava L Di Ciommo V Ronchetti MP Orzalesi M Short antibiotic prophylaxis for bacterial infections in a neonatal intensive care unit: a randomized controlled trial J Hosp Infect 2005 59 4 292 298 10.1016/j.jhin.2004.09.005 15749316

[B18] O'Grady NP Alexander M Dellinger EP Gerberding JL Heard SO Maki DG Masur H McCormick RD Mermel LA Pearson ML Guidelines for the prevention of intravascular catheter-related infections Am J Infect Control 2002 30 8 476 489 10.1067/mic.2002.129427 12461511

[B19] Mehta A Prevention and management of neonatal hypoglycaemia Arch Dis Child Fetal Neonatal Ed 1994 70 1 F54 59 discussion F59-60 10.1136/fn.70.1.F54 8117131 PMC1064069

[B20] Maindonald J Braun J Chapter 11 Data Analysis and Graphics Using R -- an Example-Based Approach 2007 Cambridge University Press 350 375

[B21] Lewis R An Introduction to Classification and Regression Tree (CART) Analysis Annual Meeting of the Society for Academic Emergency Medicine: 2000; San Francisco, California 2000

[B22] Breiman L Friedman J Olshen R Stone C Classification and Regression Trees 1984 New York: Chapman & Hall (Wadsworth, Inc.)

[B23] Bachur RG Harper MB Predictive model for serious bacterial infections among infants younger than 3 months of age Pediatrics 2001 108 2 311 316 10.1542/peds.108.2.311 11483793

[B24] Peters RP Twisk JW van Agtmael MA Groeneveld AB The role of procalcitonin in a decision tree for prediction of bloodstream infection in febrile patients Clin Microbiol Infect 2006 12 12 1207 1213 10.1111/j.1469-0691.2006.01556.x 17121627

[B25] Mishra UK Jacobs SE Doyle LW Garland SM Newer approaches to the diagnosis of early onset neonatal sepsis Arch Dis Child Fetal Neonatal Ed 2006 91 3 F208 212 10.1136/adc.2004.064188 16632649 PMC2672708

[B26] Kermorvant-Duchemin E Laborie S Rabilloud M Lapillonne A Claris O Outcome and prognostic factors in neonates with septic shock Pediatr Crit Care Med 2008 9 2 186 191 10.1097/PCC.0b013e31816689a8 18477932

[B27] Ronnestad A Abrahamsen TG Medbo S Reigstad H Lossius K Kaaresen PI Engelund IE Irgens LM Markestad T Septicemia in the first week of life in a Norwegian national cohort of extremely premature infants Pediatrics 2005 115 3 e262 268 10.1542/peds.2004-1834 15687417

[B28] Tessin I Trollfors B Thiringer K Larsson P Ampicillin-aminoglycoside combinations as initial treatment for neonatal septicaemia or meningitis. A retrospective evaluation of 12 years' experience Acta Paediatr Scand 1991 80 10 911 916 10.1111/j.1651-2227.1991.tb11752.x 1755296

[B29] Heimler R Nelin LD Billman DO Sasidharan P Identification of sepsis in neonates following maternal antibiotic therapy Clin Pediatr (Phila) 1995 34 3 133 137 10.1177/000992289503400303 7774139

[B30] Schelonka RL Chai MK Yoder BA Hensley D Brockett RM Ascher DP Volume of blood required to detect common neonatal pathogens J Pediatr 1996 129 2 275 278 10.1016/S0022-3476(96)70254-8 8765627

[B31] Kellogg JA Ferrentino FL Goodstein MH Liss J Shapiro SL Bankert DA Frequency of low level bacteremia in infants from birth to two months of age Pediatr Infect Dis J 1997 16 4 381 385 10.1097/00006454-199704000-00009 9109140

[B32] Kellogg JA Manzella JP Bankert DA Frequency of low-level bacteremia in children from birth to fifteen years of age J Clin Microbiol 2000 38 6 2181 2185 10834973 10.1128/jcm.38.6.2181-2185.2000 PMC86758

[B33] Reier-Nilsen T Farstad T Nakstad B Lauvrak V Steinbakk M Comparison of broad range 16S rDNA PCR and conventional blood culture for diagnosis of sepsis in the newborn: a case control study BMC Pediatr 2009 9 5 10.1186/1471-2431-9-5 19152691 PMC2635358

